# Conservation and diversification of the transcriptomes of adult *Paragonimus westermani* and *P. skrjabini*

**DOI:** 10.1186/s13071-016-1785-x

**Published:** 2016-09-13

**Authors:** Ben-wen Li, Samantha N. McNulty, Bruce A. Rosa, Rahul Tyagi, Qing Ren Zeng, Kong-zhen Gu, Gary J. Weil, Makedonka Mitreva

**Affiliations:** 1 Infectious Diseases Division, Department of Internal Medicine, Washington University School of Medicine, St. Louis, MO USA; 2 The McDonnell Genome Institute at Washington University, St. Louis, MO USA; 3 Department of Parasitology, Xiang-Ya School of Medicine, Central South University, Changsha, Hunan Province People’s Republic of China

**Keywords:** *Paragonimus*, *Paragonimus westermani*, *Paragonimus skrjabini*, Adult stage, Lung fluke, Comparative transcriptomics, RNA-Seq

## Abstract

**Background:**

Paragonimiasis is an important and widespread neglected tropical disease. Fifteen *Paragonimus* species are human pathogens, but two of these, *Paragonimus westermani* and *P. skrjabini*, are responsible for the bulk of human disease. Despite their medical and economic significance, there is limited information on the gene content and expression of *Paragonimus* lung flukes.

**Results:**

The transcriptomes of adult *P. westermani* and *P. skrjabini* were studied with deep sequencing technology. Approximately 30 million reads per species were assembled into 21,586 and 25,825 unigenes for *P. westermani* and *P. skrjabini*, respectively. Many unigenes showed homology with sequences from other food-borne trematodes, but 1,217 high-confidence *Paragonimus-*specific unigenes were identified. Analyses indicated that both species have the potential for aerobic and anaerobic metabolism but not *de novo* fatty acid biosynthesis and that they may interact with host signaling pathways. Some 12,432 *P. westermani* and *P. skrjabini* unigenes showed a clear correspondence in bi-directional sequence similarity matches. The expression of shared unigenes was mostly well correlated, but differentially expressed unigenes were identified and shown to be enriched for functions related to proteolysis for *P. westermani* and microtubule based motility for *P. skrjabini*.

**Conclusions:**

The assembled transcriptomes of *P. westermani* and *P. skrjabini*, inferred proteins, and extensive functional annotations generated for this project (including identified primary sequence similarities to various species, protein domains, biological pathways, predicted proteases, molecular mimics and secreted proteins, etc.) represent a valuable resource for hypothesis driven research on these medically and economically important species.

**Electronic supplementary material:**

The online version of this article (doi:10.1186/s13071-016-1785-x) contains supplementary material, which is available to authorized users.

## Background

Food-borne trematode (FBT) infections are important neglected tropical diseases (NTDs) with a global public health impact estimated at more than 665 thousand disability-adjusted life years (DALYs); paragonimiasis is arguably the most important of these because it accounts for nearly 30 % of the FBT-related DALYs [1]. Approximately 20 million people already have a *Paragonimus* infection, and almost 300 million people are at risk of becoming infected [2, 3].

More than 50 species in the genus *Paragonimus* have been described, although several could be considered synonymous [4]. Fifteen species are known to infect humans, but the *P. westermani* and *P. skrjabini* species complexes are responsible for the bulk of disease in Asia, particularly in the People’s Republic of China, which has the heaviest disease burden among 48 endemic countries [3].

The life-cycle of *Paragonimus* flukes involves complex interactions with three separate hosts [3]. Embryonated eggs expelled in the sputum or feces hatch in freshwater, releasing larvae that undergo rounds of growth and asexual reproduction in the first intermediate host, an aquatic snail. The snails, in turn, release larvae that develop into metacercariae in crustaceans. When infected crustaceans are ingested by a permissive host (typically small carnivores such as canids, felids, murids, mustelids, viverrids, etc.), metacercariae migrate out of the digestive tract and into the lung, where they mature to long-lived, hermaphroditic, sexually reproducing adults within pulmonary cysts. In contrast, metacercariae ingested by a non-permissive often fail to find the lung. They remain in an immature state and migrate through abnormal tissues including the central nervous system (CNS). *Paragonimus skrjabini*, for example, is poorly adapted to humans and often causes these ectopic infections [3].

Paragonimiasis is commonly diagnosed by detecting parasite eggs in stool or sputum. Unfortunately, the time interval between infection and oviposition is typically 65–90 days [3], and migrating parasites are capable of causing disease much sooner than this [1]. Migration of worms through the abdominal cavity can cause diarrhea, abdominal pain, fever and hives. Parasites in the lung trigger asthma- or tuberculosis-like symptoms with including cough, fever, pleural effusion, chest pain and bloody sputum. Ectopic infections in the CNS can lead to headache, visual loss, or death if left untreated [1]. Paragonimiasis is easily treated with oral praziquantel. However, diagnosis and treatment are often delayed, because of the non-specific nature of the symptoms and the lack of sensitive and reliable diagnostic methods [5].

Apart from widely used phylogenetic markers, Asian *Paragonimus* species are very poorly represented in pubic sequence repositories. In the year 2015, there were only 456 protein sequences from the genus *Paragonimus* in NCBI’s non-redundant protein database (NR). This represents a significant hindrance to the biological research that will be needed to promote the development of novel methods for diagnosis, treatment and global control of paragonimiasis. In order to address this need, we have sequenced and characterized the transcriptomes of *P. westermani* and *P. skrjabini* adult worms. Transcriptome sequencing is a well-established, efficient, and cost-effective method of gene discovery that has been used to characterize the expressed genes of trematodes and other parasites [6–8].

Thus, our study has provided insights into the biology of two *Paragonimus* species along with a wealth of novel sequence data that could be explored to test specific hypotheses relating to *Paragonimus* and other FBTs.

## Methods

### Parasite material

Freshwater crab intermediate hosts were collected to obtain parasite metacercariae. Crabs of the genus *Isolaptamon* were collected from Liuyang county (now called Baisha county), Hunan Province, China, a region specifically endemic to *P. westermani* [9]. Likewise, *Sinopotamon denticulatum* were collected from Changan county of Shanxi Province, China, a region specifically endemic to *P. skrjabini* [10]. Metacercariae were isolated from crab tissue as previously described [11]. The shells of the crabs were removed and the soft tissues were processed in 1× phosphate-buffered saline with a meat grinder. The homogenized meat was allowed to settle, and the supernatant was discarded. The sediment was rinsed several times in water, and metacercariae were collected under a dissection microscope. Species identity was confirmed by morphological examination of metacercariae and later by examination of adult parasites [12–15].

Dogs obtained from non-endemic areas and clear of existing infections were inoculated orally with 200–300 *P. westermani* or *P. skrjabini* metacercariae. Adult worms were harvested from the lungs 100 days post-infection, washed thoroughly in diethylpyrocarbonate-treated water, frozen in liquid nitrogen, and stored at -80 °C prior to use.

### RNA isolation and sequencing

A total of 5 adult *P. westermani* and 5 adult *P. skrjabini* were homogenized in 1 ml TRIzol reagent with microcentrifuge pestle, and total RNA was purified from the homogenate using a TRIzol Plus RNA Purification Kit manufacturer’s recommended protocol (Thermo Fisher Scientific, Waltham, MA, USA) and DNase-treated. Samples had very prominent 28S peaks and very small 18S peaks, with RIN values and DV200 values of 8.3 and 71 (*P. westermani*, concentration 677 ng/μl) and 7.7 and 72 (*P. skrjabini*, concentration 562 ng/μl), respectively (Additional file 1: Figure S1). Sequencing libraries were prepared from 2 μg total RNA using Illumina's TruSeq Stranded mRNA Library Preparation Kit according to the manufacturer’s protocol and sequenced on the Illumina HiSeq2000 platform (Illumina, San Diego, CA, USA). Raw reads (100 bp in length) were deposited in the NCBI sequence read archive under BioProject ID PRJNA219632 for *P. westermani* and PRJNA301597 for *P. skrjabini*.

### RNA-Seq read processing and assembly

Raw reads were subjected to stringent quality control and contaminant filtering as previously described [16]. Briefly, reads were trimmed to remove low quality regions, and filtered based on read length, sequence complexity, and similarity to known or suspected contaminants, including ribosomal RNA [17, 18], bacteria [19], *Homo sapiens* (GenBank version hs37) and *Canis familiaris* (GenBank version 3.1). Remaining high-quality, contaminant-free read sets were down-sampled by digital read normalization using khmer (k = 20) [20]. Reads selected in the down-sampling and their mates were assembled using the Trinity *de novo* RNA-Seq assembler using default parameters [21]. Scripts included in the Trinity software package were used to map the complete, cleaned read set to the assembled transcripts and filter transcripts less than 1 transcript per million reads mapped and less than 1 % of the per unigene expression level [21]. Assembly fragmentation was calculated with respect to *Clonorchis sinensis* coding sequences (WormBase ParaSite BioProject PRJDA72781) using in-house scripts and is reported as the percentage of reference genes matched to multiple, non-overlapping transcript BLAST hits.

### Transcript expression analyses

The complete, cleaned read sets were mapped to the corresponding filtered, high-quality transcript assemblies, and fragments per kilobase of exon per million mapped fragments (FPKM) were calculated for each unigene according to an RNA-Seq by expectation-maximization (RSEM) protocol using scripts included in the Trinity software package [21]. Unigenes were ranked according to abundance based on FPKM values. Fold changes were calculated for the corresponding unigenes from the two assemblies. The average fold change plus or minus 1.96 times the standard deviation (corresponding to the top 5th percentile of up-/downregulation) was used as a cut-off to select unigenes that were differentially expressed between the two species.

### Protein prediction and functional annotation

Protein sequences were predicted from transcripts using Prot4EST [22] based, in part, on results from BLAST searches against the NCBI non-redundant protein database (NR, downloaded on 15 April 2014) and databases of ribosomal [17, 18] and mitochondrial genes (downloaded from GenBank on 26 July 2013).

Protein translations were compared to known proteins in NR (downloaded on 15 June 2015), *Clonorchis sinensis* (WormBase ParaSite BioProject PRJDA72781), *Opisthorchis viverrini* [23], *Fasciola hepatica* [24] and *Paragonimus kellicotti* [16] protein sequences by BLASTP, and results were parsed to consider only non-overlapping top hits with e-value ≥ 1e^-05^. Sequences from *Paragonimus* species were excluded from NR prior to BLAST searches in order to facilitate identification of genus- and species-specific transcripts. The longest predicted protein isoform of each assembly unigene was also subjected to a reciprocal best BLAST match between the *P. skrjabini* and *P. westermani* transcripts with an e-value cut-off of 1e^-05^.

Predicted proteins were matched to conserved domains (InterPro) and gene ontology (GO) terms using InterProScan [25–27]. Associations with biological pathways (KEGG orthologous groups, pathways and pathway modules) were determined by KEGGscan [28, 29] using version 70 of the KEGG database. KEGG module completion was determined as previously described [30]. Putative proteases and protease inhibitors were identified and classified by comparison with the MEROPS database [31]. Classical secretion signals found within the first 70 N-terminal amino acids and transmembrane domains were predicted with Phobius [32]. All assembled transcripts, predicted proteins, and associated functional annotations are available at Trematode.net [33].

### Identification of “host mimic” proteins

The longest isoform of each assembly unigene was compared to proteins from *Homo sapiens* (NCBI hs38) and the closest sequenced free-living relative, *Schmidtea mediterranea* (WormBase ParaSite Bioproject PRJNA12585), by BLASTP. Deduced *Paragonimus* proteins were considered putative “host mimics” when they shared at least 70 % sequence identity over at least 50 % of the length with the human ortholog but less than 50 % identity (if any) with the *S. mediterranea* ortholog.

### Functional enrichment of gene ontology (GO) terms

Functional enrichment of GO terms was calculated using FUNC with a *P*-value cut-off of 0.01 [34]. In all cases, the target list was comprised of the longest transcript of each unigene associated with the feature of interest and the background list was comprised of the target list plus the longest transcript from each remaining unigene.

## Results and discussion

### Transcriptome sequencing, assembly and annotation

**Fig. 1:**
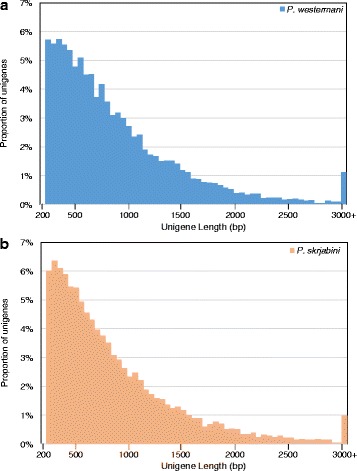
Unigene length distribution for *Paragonimus westermani* (**a**) and *Paragonimus skrjabini* (**b**)

The adult transcriptomes of *P. westermani* and *P. skrjabini* were sequenced, assembled *de novo*, and filtered to consider only high-confidence transcript sequences (Table 1). In each case, related transcripts thought to result from alternative splicing of the same gene were clustered into “unigenes”. A total of 27,842 transcripts from 21,586 unigenes were generated from *P. westermani* while 35,312 transcripts from 25,825 unigenes were generated from *P. skrjabini*. Unigenes from the two species had similar length distribution patterns (Fig. 1). We expect these species to encode a gene complement similar in size to those of other FBTs: 13,634 for *C. sinensis* [35], 16,379 for *O. viverrini* [23] and 15,740 for *F. hepatica* [24]. In an ideal assembly, the number of unigenes would equal the number of genes expressed genes in the life-cycle stage or condition studied. However, *de novo* short read assemblies tend to be fragmented, and this inflates unigene counts. Fragmentation, reported as the percentage of reference genes matched to non-overlapping transcript BLAST hits, was estimated at 24.3 % for *P. westermani* and 26.7 % for *P. skrjabini* with respect to the protein coding sequences of *C. sinensis*. For clarification, this indicates that 24.3 % of all *C. sinensis* genes are associated with multiple, non-overlapping *P. westermani* transcripts.

**Table 1:** Sequencing, assembly and annotation of the transcriptomes of adult *P. westermani* and *P. skrjabini*

	*P. westermani*	*P. skrjabini*
Sequence data		
Raw read pairs	46,468,226	49,816,749
Clean read pairs	34,096,586	38,071,235
Raw transcript assembly		
Unigenes	54,488	90,091
Transcripts	71,317	126,745
Filtered transcript assembly		
Unigenes	21,586	25,825
Transcripts	27,842	35,312
Mean unigene length (bp)	813.0 ± 598.5	772.4 ± 570.0
Mean transcript length (bp)	853.9 ± 600.1	834.8 ± 595.9
Fragmentation rate	24.3 %	26.7 %
Predicted proteins		
Unique protein translations	26,431	32,706
Unigenes	21,585	25,822
Transcripts	27,838	35,305
Mean protein length (aa)	271.0 ± 199.5	257.5 ± 190.0
Annotation (functional terms / unigenes)		
Unique InterPro domains	4,190 / 8,853	3,263 / 7,152
Unique GO terms	1,197 / 6,964	1,024 / 5,460
Unique KEGG orthologous groups	3,618 / 13,257	3,605 / 12,168
Unique KEGG pathways	313 / 8,081	313 / 7,426
Unique KEGG pathway modules	218 / 3,189	220 / 2,897

A total of 26,431 and 32,796 unique protein translations were generated from *P. westermani* and *P. skrjabini* respectively, and these were annotated based on similarity to sequences in various publicly available databases (Table 1). Complete annotations are provided in Additional file 2: Table S1 and Additional file 3: Table S2. Altogether, functional information (e.g. BLAST matches, structural domains, functional classification, etc.) was deduced for a majority of unigenes, 79.3 % and 80.0 % for *P. westermani* and *P. skrjabini*, respectively.

### Sequence conservation with relevant trematode species

**Fig. 2:**
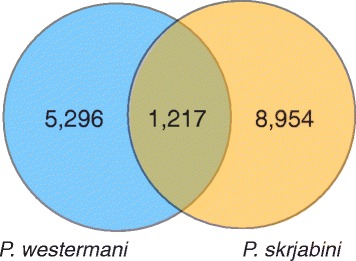
*Paragonimus*-specific proteins from *Paragonimus westermani* and *Paragonimus skrjabini*. Predicted proteins from 6,513 *P. westermani* unigenes and 10,171 *P. skrjabini* unigenes found no significant match to non-*Paragonimus* sequences in NR. Of the unigenes with no BLAST match in NR, 1,217 from each assembly were matched to a non-hit unigene in the other assembly

Due to the sparse representation of *Paragonimus* sequences in public sequence repositories, only a small fraction of our predicted proteins shared highest sequence similarity with *Paragonimus* sequences in NR (125 transcripts from 86 *P. westermani* unigenes and 151 transcripts from 88 *P. skrjabini* unigenes); a majority of these also had close matches to non-*Paragonimus* sequences. Predicted proteins from 69.8 % and 60.6 % of *P. westermani* and *P. skrjabini* unigenes, respectively, had top matches to non-*Paragonimus* proteins in NR (Additional file 2: Table S1 and Additional file 3: Table S2) due to the underrepresentation of *Paragonimus* spp. references in NR. Top hits were mostly to other food-borne trematodes, particularly *C. sinensis* and *O. viverrini*. Some 1,217 of the 6,513 *P. westermani* and 10,171 of the *P. skrjabini* unigenes with no significant match to non-*Paragonimus* proteins in NR were homologous in both species (i.e. conserved hypothetical unigenes, Fig. 2). This strengthens the notion that they are indeed valid (not caused by assembly errors), *Paragonimus*-specific transcripts.

Comparisons to other trematode species at the primary sequence level indicated that deduced proteins from *P. westermani* and *P. skrjabini* share higher sequence identity with proteins from *P. kellicotti* (the only *Paragonimus* species with an available adult transcriptome) compared to other FBTs (Table 2). *Paragonimus westermani* and *P. skrjabini* may share slightly higher sequence identity with *C. sinensis* as compared to *O. viverrini* and *F. hepatica*; however, this result may be biased by the quality and completeness of the genome assemblies and gene models included in the analysis, as phylogenetic analyses based on mitochondrial markers have previously placed *Paragonimus* alongside *F. hepatica* rather than the carcinogenic liver flukes [36, 37].

**Table 2:** BLASTP comparisons of *P. westermani* and *P. skrjabini* proteins with selected trematode species. The number of transcripts/unigenes with BLASTP match (e-value < 1e^-05^) to subject proteins is indicated. Average percent identity was calculated based on the top hit to the longest isoform of each unigene

	*P. westermani* (27,838 / 21,585)	*P. skrjabini* (35,305 / 25,822)
*Clonorchis sinensis*	18,144 / 14,164 (62.3 %)	19,271 / 14,441 (64.0 %)
*Opisthorchis viverrini*	18,489 / 14,423 (61.7 %)	20,186 / 14,974 (63.6 %)
*Fasciola hepatica*	17,246 / 13,403 (59.6 %)	17,848 / 13,308 (62.2 %)
*Paragonimus kellicotti*	19,529 / 15,075 (85.5 %)	22,493 / 16,296 (86.4 %)
*Paragonimus westermani*	–	22,496 / 16,290 (85.8 %)
*Paragonimus skrjabini*	18,890 / 14,693 (84.3 %)	–

### Metabolic potential of *Paragonimus westermani* and *P. skrjabini*

**Fig. 3:**
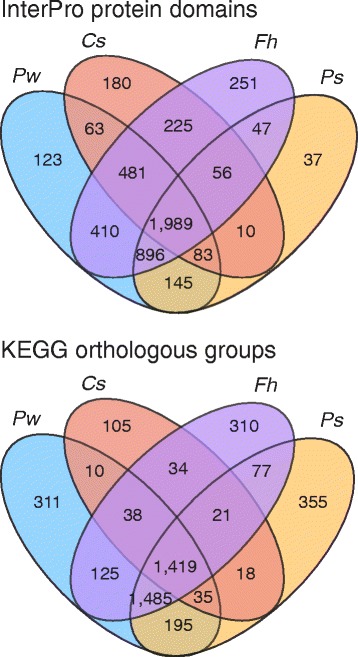
Distribution of InterPro domains and KEGG orthologous groups among selected food-borne trematodes. InterPro protein domains and KEGG orthologous groups were assigned to proteins from the complete genomes of *Clonorchis sinensis* and *Fasciola hepatica* and to proteins predicted from the *Paragonimus westermani* and *Paragonimus skrjabini* transcriptome assemblies, and intersections were determined. *Abbreviations*: *Pw*, *P. westermani*; *Cs*, *C. sinensis*; *Fh*, *F. hepatica*; *Ps*, *P. skrjabini*

Translated proteins were matched to KEGG orthologous groups and their parent unigenes were binned into broad functional categories (Table 3). The most abundantly populated categories from both assemblies were “signal transduction”, “translation” and “protein folding, sorting, and processing”. Most of the InterPro domains and KEGG orthologous groups that were represented in the adult transcriptomes of *P. westermani* and *P. skrjabini* were also represented in the genomes of other food-borne trematodes (Fig. 3). The 1,989 conserved protein domains and 1,419 conserved KOs provide a catalog of functions involved in core biological processes common to all sequenced FBTs. *Paragonimus westermani* and *P. skrjabini* shared more InterPro domains with the genome of *F. hepatica* as compared to the genome of *C. sinensis*. Some 145 InterPro domains and 195 KEGG orthologous groups were represented in the transcriptome assemblies of both *Paragonimus* species but absent from the draft genomes of the other two flukes. These *Paragonimus* conserved/specific KEGG orthologous groups were involved in 28 unique modules, all of which were sparsely populated (Additional file 4: Table S3); therefore, it is difficult to comment on metabolic differences between *Paragonimus* and other FBTs based solely on the transcriptomes.

**Table 3:** KEGG categorization of assembled unigenes

	*P. westermani*	*P. skrjabini*
Cellular processes	1,709	1,645
Cell communication	504	512
Cell growth and death	561	495
Cell motility	239	220
Transport and catabolism	795	787
Environmental information processing	1,455	1,372
Membrane transport	72	74
Signal transduction	1,306	1,220
Signaling molecules and interaction	159	157
Genetic information processing	3,303	3,011
Folding, sorting and degradation	1,142	1,087
Replication and repair	460	361
Transcription	775	706
Translation	1,208	1,138
Metabolism	2,407	2,223
Amino acid metabolism	425	395
Biosynthesis of other secondary metabolites	59	54
Carbohydrate metabolism	539	514
Energy metabolism	428	402
Glycan biosynthesis and metabolism	316	304
Lipid metabolism	386	353
Metabolism of cofactors and vitamins	246	216
Metabolism of other amino acids	185	169
Metabolism of terpenoids and polyketides	102	90
Nucleotide metabolism	391	375
Xenobiotics biodegradation and metabolism	87	104

The metabolic potential of the two species was assessed at the level of KEGG pathway modules. A KEGG module is considered to be complete when the transcriptome includes the full complement of enzymes (assessed at the level of KO’s) necessary to convert the initial substrate to the final product. Of 95 helminth-relevant KEGG modules [30], 35 were complete in both *P. westermani* and *P. skrjabini.* A total of 30 complete modules are shared between the two, with five uniquely complete in each species. However, the difference between the complete modules in one species and the incomplete modules in the other is at most two KO’s, suggesting high functional conservation among the two species.

Other FBTs are known to undergo transitions in energy metabolism over the course of the life-cycle, shifting from aerobic respiration in larval stages to anaerobic respiration in adult stages to adapt to low oxygen microenvironments in host tissues [23, 24, 38]. Given that oxygen tension fluctuates within parasite lung cysts, adult *P. westermani* are thought to be facultative anaerobes with separate populations of mitochondria capable of either aerobic or anaerobic respiration [39, 40]. Pathway modules associated with aerobic respiration (e.g. M00087: beta-oxidation, M00009: citrate cycle, M00148: succinate dehydrogenase, etc.) were complete in both transcriptomes (Additional file 4: Table S3), and key enzymes involved in anaerobic dismutation (e.g. phosphoenolpyruvate carboxykinase) were also identified (Additional file 2: Table S1 and Additional file 3: Table S2). Modules related to fatty acid initiation (M00082, two of 13 KOs) and elongation (M00083, one of 14 KOs) are incomplete and poorly represented, so it is unlikely that these processes take place in adult *Paragonimus* (Additional file 4: Table S3), although fatty acid binding proteins were identified in both species (based on NR matches; comp22449_c0 and comp19053_c0 in *P. westermani* and comp74673_c0 in *P. skrjabini*). This is consistent with the hypothesis that trematodes (with the possible exception of *C. sinensis* [35]) are incapable of *de novo* fatty acid biosynthesis [23, 24, 41].

### Host-parasite interaction

Secreted and excreted proteins are of particular interest in parasites like *Paragonimus*. They often play important roles in host parasite interaction [41, 42] and are useful targets for diagnostic assays [43–45]. While the N-terminal regions of proteins, which contain secretion signals, are often underrepresented in transcriptome assemblies, 622 *P. westermani* and 750 *P. skrjabini* unigenes were found to contain classical signal peptides and no transmembrane domains. This suggests that they may be secreted from cells. Several GO terms related to proteolysis and redox regulation were enriched in the putative secreted unigenes in both species (Additional file 5: Table S4). This is consistent with previous findings that highlighted the prevalence of proteases in trematode excretory-secretory products [46–49] and outlined their important roles in migration through host tissues, feeding and immune evasion [50–53].

Molecular mimicry is a well-known strategy for host manipulation and immune evasion [54]. Interestingly, 122 and 134 predicted proteins from *P. westermani* and *P. skrjabini* had far better blast matches to *Homo sapiens* (a potential host species) than to the free-living, freshwater planarian platyhelminth, *Schmidtea mediterranea* (Additional file 2: Table S1; Additional file 3: Table S2; see Methods for details). These putative “host mimic” proteins were enriched for kinase and GTPase activity in both species (Additional file 5: Table S4), which may indicate roles in signaling. Parasites like *Plasmodium* spp., *Echinococcus multilocularis* and *Schistosoma mansoni* are known to possess functional homologs of host hormone receptors [54–57]; thus there is a precedent for comingling of host and parasite signaling pathways.

### Gene expression in *Paragonimus westermani* and *P. skrjabini*

**Fig. 4:**
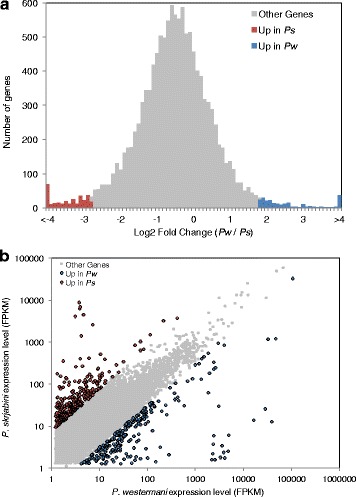
Estimated expression of *Paragonimus westermani* and *P. skrjabini* assembly unigenes. Corresponding *P. westermani* and *P. skrjabini* unigenes identified by bi-directional blast search. The expression levels of each unigene (FPKM) were estimated, and the expression of matched unigenes were compared. **a** The average fold change plus or minus 1.96 times the standard deviation (corresponding to the top 5th percentile of differential regulation) was used as a cutoff to select unigenes differentially expressed between the two species. **b** Expression levels of corresponding unigenes were plotted, and differentially expressed unigenes are colored

Expression levels were estimated for each unigene in the two transcriptome assemblies (Additional file 2: Table S1; Additional file 3: Table S2). As expected, the top 5 % most highly expressed unigenes in both assemblies were enriched for GO terms related to basic cellular functions such as translation, ATP synthesis and redox regulation (Additional file 5: Table S4). Finding a direct one-to-one correlation between assembly unigenes can be challenging due to the incompleteness and fragmentation of *de novo* transcript assemblies; however, 12,432 *P. westermani* and *P. skrjabini* unigenes were linked through a bi-directional blast match of the longest transcript isoform from each. The expression of matched unigenes tended to be well correlated, but some differentially expressed unigenes were identified (Fig. 4, Table 4). The 303 unigenes that were upregulated in *P. westermani* were enriched with GO terms related to endopeptidase activity whereas the 249 unigenes upregulated in *P. skrjabini* were enriched with GO terms related to microtubule based movement (Additional file 5: Table S4). Disparities in gene complement and expression such as these could account for the striking biological differences between *P. westermani* and *P. skrjabini.*

**Table 4:** Top 30 differentially expressed unigenes of *P. westermani* and *P. skrjabini*

*P. westermani* unigene	*P. skrjabini* unigene	Top non-*Paragonimus* NR BLASTP match of upregulated unigene (e-value)	*P. westermani* FPKM	*P. skrjabini* FPKM	Fold change (*P. westermani* / *P. skrjabini*)
comp24563_c2	comp79108_c0	*C. sinensis* cysteine protease (5e^-81^)	39,412.97	12.39	11.64
comp20235_c3	comp97009_c0	*C. sinensis* cysteine protease (2e^-81^)	4,150.67	1.72	11.24
comp18122_c0	comp67960_c0	*Pelodiscus sinensis* papain-like protein (7e^-42^)	4,831.75	2.47	10.93
comp26350_c1	comp73392_c0	*F. hepatica* ferritin-like protein (8e^-26^)	3,368.34	1.79	10.88
comp22100_c0	comp49569_c0	*Haplorchis taichui* cytochrome *c * oxidase subunit III (8e^-45^)	3,992.12	2.35	10.73
comp23043_c0	comp67491_c0	*Strongylocentrotus purpuratus* proactivator polypeptide (4e^-12^)	2,567.33	1.55	10.69
comp22520_c0	comp77222_c0	*C. sinensis* cathepsin F precursor (9e^-82^)	2,189.89	1.59	10.43
comp26308_c0	comp83382_c0	*Fasciola gigantic*a legumain-1 (4e^-102^)	16,728.14	13.71	10.25
comp23905_c0	comp85010_c1	*Amphimedon queenslandica* uncharacterized protein (1e^-08^)	29,015.14	25.29	10.16
comp24308_c0	comp80461_c0	*Trichobilharzia regenti* cathepsin B1 isotype 1 precursor (2e^-15^)	3,638.33	3.32	10.10
comp19972_c0	comp81354_c0	*Fasciola* sp. cytochrome *c* oxidase subunit 2 (3e^-70^)	2,334.18	2.22	10.04
comp25450_c1	comp80216_c0	*O. viverrini* hypothetical protein (3e^-30^)	4,984.93	5.99	9.70
comp26673_c2	comp57972_c0	*S. mansoni* reverse transcriptase (1e^-80^)	2,399.15	3.70	9.34
comp19193_c2	comp66866_c0	*O. viverrini* hypothetical protein (1e^-60^)	2,431.20	5.89	8.69
comp27091_c0	comp77961_c0	*C. sinensis* hypothetical protein (4e^-101^)	286.23	2.07	7.11
comp16826_c0	comp85805_c0	*C. sinensis* ELAV like protein (4e^-48^)	2.13	130.95	-5.94
comp19146_c1	comp87478_c0	*C. sinensis* malate dehydrogenase (2e^-37^)	6.19	407.90	-6.04
comp13982_c0	comp90793_c1	*S. mansoni* reverse transcriptase (3e^-26^)	2.03	139.37	-6.10
comp29521_c0	comp86043_c1	–	3.25	255.19	-6.29
comp17946_c0	comp90932_c0	–	1.58	150.89	-6.58
comp19442_c0	comp86832_c0	*F. hepatica* mitochondrial acetate:succinate CoA-transferase (1e^-17^)	4.02	395.13	-6.62
comp65706_c0	comp89911_c1	–	1.36	174.31	-7.00
comp18789_c0	comp86285_c0	*O. viverrini* hypothetical protein (2e^-23^)	1.82	346.66	-7.57
comp63230_c0	comp84630_c0	–	4.22	1,369.68	-8.34
comp7414_c0	comp80611_c0	*O. viverrini* hypothetical protein (5e^-34^)	7.50	2,512.29	-8.39
comp29913_c0	comp84815_c0	–	4.75	4,443.78	-9.87
comp6439_c0	comp78453_c1	*Tetrancistrum nebulosi* cytochrome *c* oxidase subunit III (2e^-17^)	3.18	4,014.38	-10.30
comp14255_c0	comp79740_c0	–	3.97	6,073.01	-10.58
comp14876_c0	comp83945_c0	*Acyrthosiphon pisum* kunitz-type proteinase inhibitor (3e^-14^)	4.09	6,662.22	-10.67
comp16223_c0	comp82281_c1	–	3.83	8,683.69	-11.15

### Diagnostic potential of deduced *P. westermani* and *P. skrjabini* proteins

**Fig. 5:**
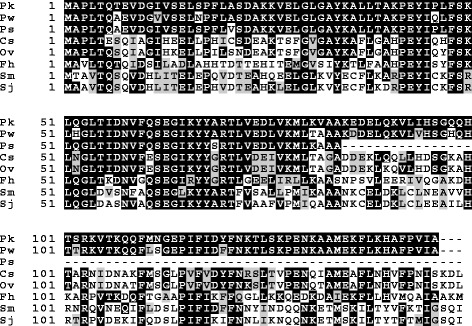
Alignment of myoglobin orthologs from *Paragonimus* species and other trematodes. Although assembly fragmentation resulted in a truncated sequence from *P. skrjabini*, it had greater 90 % similarity with *Paragonimus* myoglobin (at the amino acid level), with much less similarity to myoglobins from other trematodes. *Abbreviations*: Pk, Pk34178_txpt1 [16]; Pw, comp20873_c0_seq2; Ps, comp80973_c0_seq3; Cs, *C. sinensis* gi:349998765; Ov, *Opisthorchis viverrini* gi: 663047528; Fh, *F. hepatica* gi:159461074; Sm, *S. mansoni* gi:256084837; Sj, *S. japonicum* gi:226487206

In a previous study, proteins predicted from the *de novo* transcriptome of *P. kellicotti* were used as a comparative database in a mass spectrometry study aimed at identifying parasite proteins that could be used as serodiagnostic markers [16]. *Paragonimus kellicotti* proteins were immunoaffinity-purified from worm lysate with IgG from the serum of infected patients and proteins predicted from 321 transcripts (227 unigenes) were identified by mass spectrometry. Some 205 of the immunoreactive *P. kellicotti* proteins have blast matches to proteins deduced from the transcriptomes of both *P. westermani* and *P. skrjabini* (Additional file 2: Tables S1; Additional file 3: Table S2). Among these conserved proteins was a putative myoglobin isoform proposed as a diagnostic candidate due to its high detection levels in the MS study and its low sequence conservation with trematodes of other genera (Fig. 5). Further studies will be needed to thoroughly explore the utility of this protein as a pan-*Paragonimus* diagnostic marker.

## Conclusions

This study provides the first insights into gene content and expression in *P. westermani* and *P. skrjabini*. Genetic conservation and diversification were assessed to characterize present and absent metabolic pathways. Like other FBTs [23, 24, 41]*,* these species appear capable of both aerobic or anaerobic metabolism, but not *de novo* fatty acid biosynthesis. For the most part, conserved unigenes were expressed to similar degree in both species. Genes upregulated in *P. westermani* were enriched for GO terms related to proteolysis while genes upregulated in *P. skrjabini* were enriched for GO terms related to microtubule based movement. Expressed orthologs of *P. kellicotti* serodiagnostic antigens were identified in both species, and should be explored in pan-*Paragonimus* diagnostic assays. We expect that the assembled transcriptomes and the accompanying functional annotations will be a valuable resource for future research, including ongoing genome sequencing projects [33].

## Additional files

Additional file 1: Figure S1. Quality metrics for RNA samples used in the RNA-Seq experiment. Electrophoresis results and RIN graphs are included for (A) *P. westermani* and (B) *P. skrjabini*. (TIF 372 kb) Additional file 2: Table S1. Complete functional annotation and expression data for *P. westermani* transcripts. (XLSX 6726 kb) Additional file 3: Table S2. Complete functional annotation and expression data for *P. skrjabini* transcripts. (XLSX 7927 kb) Additional file 4: Table S3. KEGG module representation and completeness for *P. westermani* and *P. skrjabini*. (XLSX 49 kb) Additional file 5: Table S4. Gene Ontology term enrichment among transcript sets of interest from *P. westermani* and *P. skrjabini*. (XLSX 42 kb)

## Abbreviations

FBT: Food-borne trematode
FPKM: Fragments per kilobase of exon per million fragments mapped
GO: Gene ontology
NR: NCBI’s non-redundant protein database
RSEM: RNA-Seq by expectation maximization
