# Supplementary figures and images for: Conservation and diversification of the transcriptomes of adult Paragonimus westermani and P. skrjabini
Source: Parasit Vectors. 2016 Sep 13;9(1):497. doi: 10.1186/s13071-016-1785-x (PMC5020434; doi:10.1186/s13071-016-1785-x)

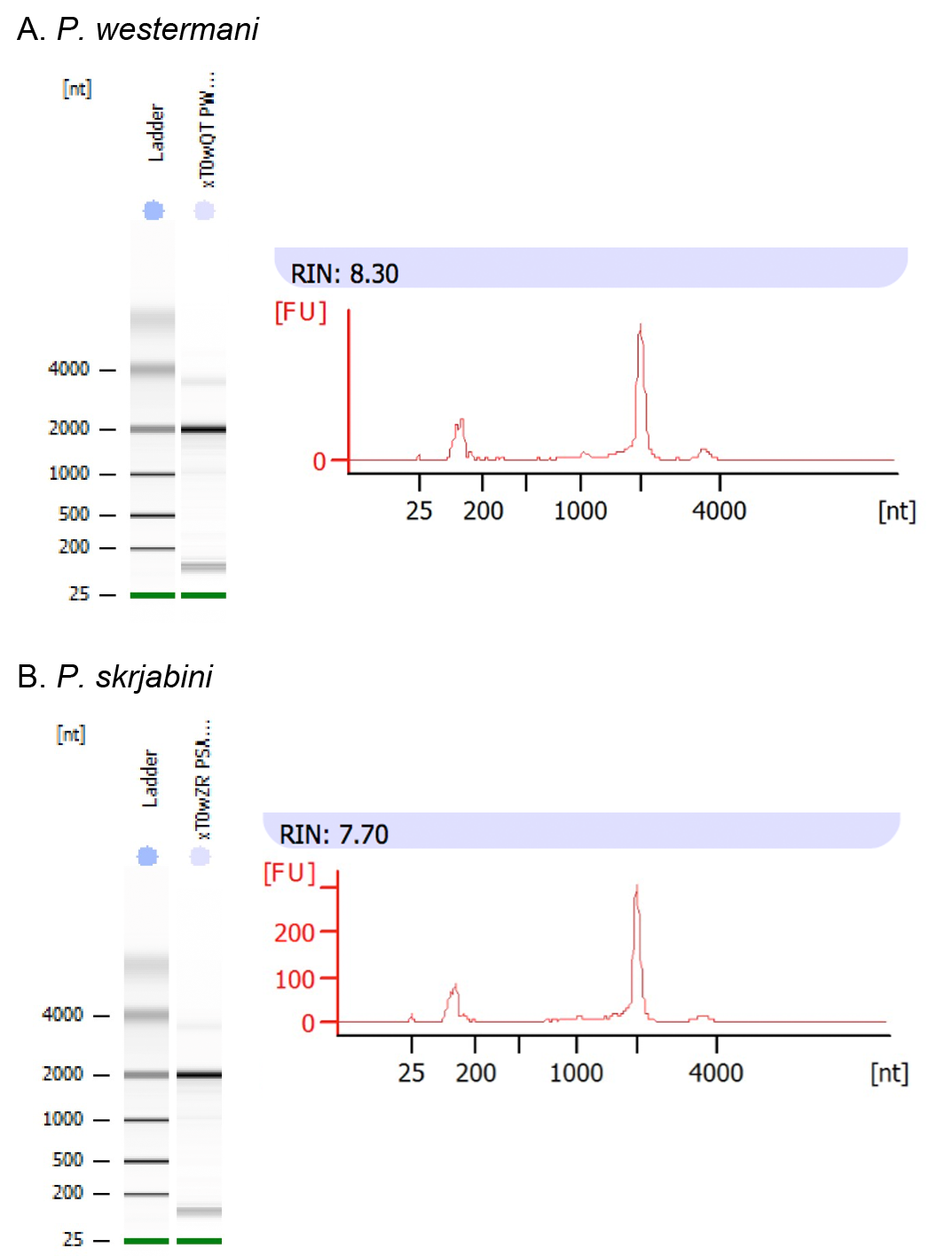

Supplement: Additional file 1: Figure S1. — Quality metrics for RNA samples used in the RNA-Seq experiment. Electrophoresis results and RIN graphs are included for (A) P. westermani and (B) P. skrjabini. (TIF 372 kb) [file 13071_2016_1785_MOESM1_ESM.tif]
